# Retraction: *In situ* Raman investigation of the phase transition of NaVO_2_F_2_ under variable temperature conditions

**DOI:** 10.1039/d2ra90066a

**Published:** 2022-07-19

**Authors:** Sa Zhang, Yan Li, Liuqing Huang, Liuying Huang, Xuetao Luo

**Affiliations:** College of Materials, Department of Materials Science and Engineering, Xiamen University Xiamen Fujian Province 361005 China liyan@stu.xmu.edu.cn; Fujian Key Laboratory of Advanced Materials, Xiamen University Xiamen Fujian Province 361005 China

## Abstract

Retraction of ‘*In situ* Raman investigation of the phase transition of NaVO_2_F_2_ under variable temperature conditions’ by Sa Zhang *et al.*, *RSC Adv.*, 2021, **11**, 23550–23556, https://doi.org/10.1039/D1RA02827H.

We, the named authors, hereby wholly retract this *RSC Advances* article due to issues with the equipment used during the research and the resulting errors due to an unstable temperature control system.

A Raman spectrometer was used for the testing outlined in this research, utilising coupled variable temperatures to investigate the phase transition of NaVO_2_F_2_. The temperature change was controlled by a THMS600 high and low-temperature sample stand. After the publication of this research, the unstable temperature was found to be caused by the system of the THMS600 sample stage, which lead to the temperature error in the analysis of phase transformation during the heating process. This has led to errors in the analysis, and therefore the conclusions of this paper are not supported. The authors also regret that the funding information was incorrectly shown in the original article.

Signed: Sa Zhang, Yan Li, Liuqing Huang, Liuying Huang and Xuetao Luo, 23rd June 2022.

Retraction endorsed by Laura Fisher, Executive Editor, *RSC Advances*.

## Supplementary Material

